# Development and validation of Japanese version of alternative food neophobia scale (J-FNS-A): association with willingness to eat alternative protein foods

**DOI:** 10.3389/fnut.2024.1356210

**Published:** 2024-05-28

**Authors:** Mio Kamei, Misaki Nishibe, Fuyumi Horie, Yuko Kusakabe

**Affiliations:** Food Research Institute, National Agriculture and Food Research Organization, Tsukuba, Ibaraki, Japan

**Keywords:** food neophobia, alternative protein, entomophagy, clean meat, lab-grown meat

## Abstract

**Introduction:**

Food neophobia (FN) is a psychological trait that inhibits one’s willingness to eat unfamiliar foods. It is related to the acceptance of insect foods and cultured meat, which are major protein alternatives to conventional meat, and is an important personality trait for understanding the near-future food industry. However, the factor structure of Pliner and Hobden’s FN scale (FNS) is unstable due to respondents’ cultural backgrounds. Thus, we aimed to develop a Japanese version based on the alternative FNS (FNS-A), the most recent revised version, and to examine its validity.

**Methods:**

Four online surveys (preliminary 1: *n* = 202; preliminary 2: *n* = 207; main: *n* = 1,079; follow-up: *n* = 500) were conducted on the FNS-A. For the main survey, Japanese respondents (aged 20–69 years) answered the Japanese version of the FNS-A (J-FNS-A), their willingness to eat (WTE), and their familiarity with hamburgers containing regular protein foods (ground beef, tofu) and alternative protein foods (soy meat, cultured meat, cricket powder, algae powder, and mealworm powder).

**Results:**

Consistent with the FNS-A, confirmatory factor analysis assuming a two-dimensional structure (approach and avoidance) showed satisfactory model fit indices. The mean J-FNS-A score (Cronbach’s α for 8 items = 0.83) was 4.15 [standard deviation (*SD*) = 0.93]. J-FNS-A scores were not associated with age and gender, whereas a greater than moderate association was found with WTE hamburgers containing alternative protein foods (*r*s = −0.42 to −0.33). The strength of these negative associations increased as food familiarity decreased (*r* = 0.94). The test–retest reliability at 1 month was also satisfactory (*r* = 0.79).

**Discussion:**

The validity of the J-FNS-A was confirmed. Higher J-FNS-A scores (mean = 41.51, *SD* = 9.25, converted to Pliner and Hobden’s FNS score) of the respondents suggest that Japanese people prefer conservative foods. This scale could predict the negative attitudes toward foods with low familiarity, such as alternative proteins. The J-FNS-A appears to be a useful psychological tool for assessing Japanese food neophobia tendencies and predicting novel food choices of Japanese individuals.

## 1 Introduction

The global population is projected to reach 8.5 billion in 2030 and 9.7 billion in 2050 (1), and people in low- and middle-income countries are becoming more carnivorous in recent decades. Specifically, global meat consumption *per capita* increased by 25% from 1990 to 2010 and is projected to increase by approximately 14% from 2021 to 2030 (2, 3). Population growth and increasing global meat consumption are driving the demand for meat and threatening food security (3). Furthermore, the development of the livestock industry contributes to global environmental impacts, particularly in terms of carbon and water footprints (4–6), and raises concerns regarding animal welfare (7). Addressing these challenges necessitates a shift in dietary styles to reduce the consumption of conventional meat through vegan, vegetarian, and flexitarian diets (8, 9), as well as the need to shift the use of protein foods from conventional meat to alternative sustainable resources (10, 11) or to more sustainable livestock production systems such as silvopasture, woodland, and rotational grazing (12).

A recent review by Onwezen et al. (13) focused on plant-based soy protein (soy meat) and pulses, insect foods, microalgae proteins, and cultured meat derived from beef muscle cells, as major alternative protein foods, from the three perspectives of novelty, desirability, and plausibility (14) and discussed global consumption trends and preferences (13). Increasing acceptance is crucial for the successful diffusion of novel foods, such as alternative protein foods, that have not traditionally been on the market. In addition to safety, cost, convenience, and the sensory qualities of foods play a pivotal role in their acceptability (13, 15–20). However, sensory preferences for alternative protein foods are expected or recognized to be less favorable than those for conventional meat (beef in many cases), and many alternative protein foods are not well accepted (13, 16, 21–25).

In addition to food-related factors (safety, cost, convenience, and sensory qualities), consumer psychological factors have been identified as potential inhibitors of the acceptance of alternative protein foods (4, 26, 27). Food neophobia (FN) is a major psychological factor related to the acceptance of alternative protein foods. It explains the reluctance to consume unfamiliar foods (28–30). The food neophobia scale (FNS), developed by Pliner and Hobden, allows for the quantification of FN tendencies (31) and has been translated into several languages [e.g., Brazilian Portuguese, Chinese, Finnish, French, German, Korean, Spanish, Swedish; see systematic review by Rabadán and Bernabéu (32)] and is used in research worldwide. Strong FN tendencies are associated with low dietary variety (e.g., fruit and vegetable intake), many disliked foods, low willingness to try new foods, and negative attitudes toward foods from other cultures (33–36). Moreover, there are similar concepts derived from FN, such as motivation to eat new foods (MENF) and food technology neophobia (FTN). MENF represents the willingness to try novel foods in two dimensions (approach and avoidance), which can be measured by the MENF scale (29), which may be a more detailed version of the FNS. In addition, FTN represents fear of novel food technology and can be measured by the FTN scale (FTNS) (37). FTNS scores are more than moderately associated with willingness to try current food technologies (e.g., pasteurization, high-pressure processing, modified atmosphere packaging) and novel food technologies (e.g., triploidy, genetic modification, bioactives). These suppressed behaviors are believed to be based on an organism’s survival strategy to protect itself from foods containing allergens and pathogens, and it is believed that strong anxiety and aversion to unfamiliar foods or food technology evoke rejection or avoidance of eating (38, 39).

Many alternative protein foods developed in recent years are unfamiliar to most consumers, as their ingredients and manufacturing processes differ significantly from those of conventional foods. Consequently, FN has been shown to significantly influence eating experiences and/or willingness to eat (WTE) alternative protein foods (40–42). FN has been investigated as an important psychological factor predicting the acceptance of alternative protein foods, which are expected to become more popular, as well as ways to avoid their negative effects and improve eating behavior (43). However, the concept of FN is rooted in Western culture; in East Asian countries, fewer studies have been translated into their own language (e.g., Chinese, Korean, Japanese) (34, 44) and conducted to evaluate the negative impact of FN on the acceptance of alternative protein foods (15, 33) than in Western countries.

To estimate the future diffusion of alternative protein foods in Japan, it is essential to measure Japanese FN. However, studies on Japanese FN are insufficient in terms of both quality and quantity. Even though the English version of the FNS (25) was used with Japanese participants, it lacked language information and translated content and did not assess the internal consistency of the scale or remove certain items (45–49). In the study of Imada and Yoneyama, 14-item Japanese statements related to food neophilia (3 items) and FN (11 items) were selected from 41 statements that were originally generated by 27 Japanese university students, and a factor structure of the Japanese version of the FNS was tested using an exploratory factor analysis (EFA) to verify its validity (50); however, some questions remain, such as 3 items related to the avoidance factor that do not show negative factor loadings for the approach factor. Despite this theoretical paradox, the 14-item score was used as the FN tendency in later studies (51, 52). Alternatively, removing 3 items (two of which were related to the avoidance factor) confirmed a sufficient internal consistency to use as the food neophilia scale (15). This ambiguity in scale content and item differences may lead to difficulties in comparing and integrating the findings on Japanese FN. Even in recent FN research reviews, studies on Japanese populations have not been addressed at all (32, 43).

The origin of these issues may be attributed to differences in culture and time periods. For example, Pliner and Hobden’s FNS was developed based on a survey of Canadian university students in the 1990s. Typically, items comprising recently developed psychological scales are aligned with the cultural context of the country in which they were developed, and translation into multiple languages carries the risk of altering their meaning for participants from other cultures (53). Indeed, the FNS has been modified for use in different countries and cultures by replacing words (33, 54, 55) and/or removing certain items (56–59). FN tendencies have weakened worldwide over the past two decades. This is attributed to the impact of globalization, which has increased exposure to food cultures in other countries through international travel, international trade, and restaurants serving foreign cuisine (32). In light of these cultural differences and changing times, certain items in the original FNS parameters, such as “Ethnic food looks too weird to eat” and “I like to try new ethnic restaurants” may no longer be appropriate today. To address these issues, De Kock et al. developed an alternative FNS (FNS-A) by revising and reorganizing the items of the original FNS and excluding items related to respondents’ cultural backgrounds (60). Even though the FNS-A included data from students from non-native English-speaking areas (South Africa, Botswana, and Lesotho), the FNS-A has the following key advantages: it confirms factor structure validity through factor analyses (exploratory and confirmatory); it demonstrates reliability (internal consistency and test–retest reliability); it demonstrates construct validity by testing its association with other psychological scales related to the FNS concepts (modified version of the FNS, MENF scale, and FTNS); and it can confirm its predictive validity by testing its association with liking or willingness to try unfamiliar or novel foods.

In light of the above, there is no sufficiently validated psychological scale for quantifying FN tendency in Japanese individuals. Nevertheless, it is expected that a Japanese version of the FNS-A (J-FNS-A) with sufficiently high validities (e.g., factor structural validity, internal consistency, test–retest reliability, construct validity, and predictive validity) will be developed by following the FNS-A, the most recent revised version. The development of a validated J-FNS-A would provide a quantitative assessment of the Japanese FN tendency and allow for a comparison with the results of FN studies around the world. Therefore, the purpose of this study was to develop a J-FNS-A and assess its validity among Japanese participants. The predictive validity of the J-FNS-A was assessed by testing its association with WTE novel foods (e.g., hamburgers containing alternative protein foods as patties) (22, 60–64).

## 2 Materials and methods

Before starting the survey, we obtained permission to translate the FNS-A into Japanese and use it in academic research from the two authors (corresponding and last) of the FNS-A (60). Our study consisted of two preliminary surveys (250 participants each; final number of participants in preliminary 1: *n* = 202; in preliminary 2: *n* = 207) to verify the validity of the translation of the FNS-A into Japanese, a main survey (1,500 participants; final number of participants in the main survey: *n* = 1,079) to verify the validity of the factor structure of the J-FNS-A and the predictive validity of WTE novel foods, and a follow-up survey (500 participants, final number of participants in the follow-up survey: *n* = 500) to test the reliability of the retests.

### 2.1 Participants

All surveys were conducted using an online questionnaire to recruit respondents from a wide range of age groups and a broad geographical area in Japan [excluding Nagano, where the entomophagy culture is flourishing, (47)]. All the respondents were recruited through a web-based survey company (iBridge Corp., Osaka, Japan). For each of the two preliminary surveys (surveys 1 and 2), 250 Japanese individuals aged 20–69 years (50 individuals in each age group: 20–29; 30–39; 40–49; 50–59; 60–69 years; with an equal gender ratio) were recruited. In the main survey, 1,500 Japanese individuals aged 20–69 years (300 in each age group: 20–29; 30–39; 40–49; 50–59; 60–69 years; with an equal gender ratio) were recruited. In addition, a follow-up survey was conducted 1 month later with 500 Japanese individuals aged 20–69 years (100 in each age group: 20–29; 30–39; 40–49; 50–59; 60–69 years; with an equal gender ratio) responding to the main survey. All respondents were identified by their identity documents, and there were no repeat responses in the preliminary and main surveys.

All surveys were conducted according to the principles of the Declaration of Helsinki. Prior to participation, potential participants received a brief description of the survey and were informed of their right to withdraw at any time. Informed consent was obtained from all participants through an online platform. The survey protocols were exempt from review by the Ethics Committee of the Food Research Institute, National Agriculture and Food Research Organization, Japan, for the following reasons: the survey results must be collected in an anonymous format that does not allow the identification of individuals. The content of the questions should be such that they cause (almost) no psychological stress.

### 2.2 Preliminary surveys (1 and 2)

To guarantee a reliable translation of the original questionnaire, a double-back translation (English → Japanese → English) of the 8 items constituting the FNS-A (60) was performed using an English editing service (Crimson Interactive Pvt. Ltd.). Double-back translation consisted of three steps: translation from English to Japanese, translation from Japanese to English, and verification of each translation. These three steps were each performed by three bilingual Japanese–English speakers independent from the authors. In addition, the two authors of the FNS-A (60) were then requested to check the translations. After obtaining agreement, the first preliminary survey of the J-FNS-A, including two directed question scale (DQS) items (65), was conducted, and the item–remainder (I-R) correlation coefficients were calculated for each of the 4 items and the corresponding factors (approach and avoidance). Results (valid responses: *n* = 202, including 93 men and 109 women) showed that the Pearson’s correlation coefficient for 1 item (“New foods mean an adventure for me,” 新しい食べ物は、自分にとって冒険だ。in Japanese) was quite small (*r*_approach_ = 0.13), unlike other items (*r*s_approach_ > 0.55, *r*s_avoidance_ > 0.58). Therefore, after discussing the issue with the two authors (60) mentioned above, “New foods mean an adventure for me” was revised to “Eating new food is an exciting event for me” A second preliminary survey was then conducted (valid responses: *n* = 207, 97 men and 110 women), and greater than large I-R correlation coefficients (*r*s_approach_ > 0.69, *r*s_avoidance_ > 0.53) were found for each item. The internal consistency of the 8 items was also sufficiently high (Cronbach’s α = 0.83).

### 2.3 Main and follow-up surveys

The main survey consisted of three sections: the J-FNS-A (section 1), familiarity (section 2), and WTE (section 3), each of which contained protein foods common in Japan and major alternative protein foods that are well known or expected to become popular worldwide. All participants answered sections 1, 2, and 3. The order of all items within each section was randomized (60). In section 1, participants were asked to respond to 8 items of the J-FNS-A. Participants rated the degree of agreement for each item on a 7-point Likert scale (strongly disagree = 1; neither disagree nor agree = 4; strongly agree = 7). In section 2, participants were asked to indicate their familiarity with each of the seven types of hamburgers containing different types of protein foods, such as ground beef, tofu, soy, cultured meat, cricket powder, mealworm powder, and algae powder. Participants rated familiarity using a 6-point scale [not at all familiar (do not know what this ingredient is) = 1, very familiar (eat this burger often, make it often) = 6]. In section 3, participants were asked to respond to questions about their WTE hamburgers containing the same protein food types as in section 2. Participants rated their WTE using a 6-point scale [not at all motivated to eat (definitely do not want to eat) = 1, very motivated to eat (definitely want to eat, definitely want to try) = 6]. Detailed explanation of alternative protein foods and definition of hamburger for participants presented in the web-based survey can be found in the Supplementary Table 1. Two DQS items (including “Please do not press the button and move on to the next item” and “Please select the first option from the right”) were included in each section, and responses that violated the instructions were considered to satisfice.

The follow-up survey was conducted 1 month after the main survey and consisted of only 8 items of the J-FNS-A with 2 DQS items.

### 2.4 Analysis

In the main survey, 421 participants (28.1% of the total sample) were excluded from the analysis because of one or more missing responses, two or more inappropriate DQS responses, or uniform responses in more than 90% of the total sample. The remaining 1,079 participants (505 men and 574 women, mean age = 45.79 years, standard deviation [*SD*] = 13.44) were included in the subsequent analyses. The final sample size remained large after exclusion due to the need to obtain data from a diverse population in Japan. Therefore, the sensitivity of the detection power was calculated using G*Power 3.1 (66, 67) for the correlation test (point biserial model) before starting the analysis. The sensitivity power calculation with a sample size of *n* = 1,079, α = 0.05, and 1-β = 0.95 estimated a required effect size of ρ > 0.11. The main analyses were conducted to test the validity of the factor structure of the J-FNS-A, compare the familiarity and WTE hamburgers among the different protein food types, and test the predictive validity of the J-FNS-A on WTE hamburgers containing alternative protein foods.

After checking the data distribution (floor and ceiling effects) for each item and the correlation coefficients between items in the J-FNS-A (68), a confirmatory factor analysis (CFA) was conducted to assess the factor structure validity using maximum likelihood estimation following the two-factor structural model (approach and avoidance) (60). The goodness-of-fit index (GFI), comparative fit index (CFI), root mean square error of approximation (RMSEA), and standardized residual mean of squares (SRMR) were calculated as model fit indices. Each of the 4 items corresponding to the approach was inverted in the calculation of the 8-item J-FNS-A score and in the assessment of internal consistency. After assessing the internal consistency using Cronbach’s α (69), the mean scores were calculated as FN scores. In accordance with the original FNS-A, after each of the four approach factor items was inversely calculated and combined with the avoidance factor items, the 8-item J-FNS-A (mean: 8 items) score was used for the main analysis. Higher scores indicate a stronger food neophobic tendency. The association between the 8-item J-FNS-A scores and age (20–69 years) was assessed using Pearson correlation analysis. In addition, the gender difference in the 8-item J-FNS-A score was assessed by Welch’s *t*-test.

After visually checking each food residual distribution using a normal Q–Q plot, familiarity with and WTE hamburgers containing different protein food types were compared among different protein food types (ground beef, tofu, soy meat, cultured meat, cricket powder, algae powder, and mealworm powder) using one-way repeated measure analyses of variance (ANOVA). Huynh–Feldt correction and Shaffer’s post-hoc analysis were performed when appropriate. Furthermore, the similarity of WTE between protein foods was assessed using polycholic correlation analysis. In addition, as a food-level analysis, a Pearson correlation analysis was conducted between the WTE hamburgers (mean of each protein food) and familiarity (mean of each protein food). The association between WTE and age was assessed using polyserial correlation analysis. In addition, the gender difference in WTE for each hamburger was assessed by Welch’s *t*-test.

The predictive validity of the J-FNS-A was assessed by calculating the associations between J-FNS-A scores and WTE hamburgers containing different protein foods as patties using polyserial correlation analysis. In addition, to understand the strength of the associations (correlation coefficients between the J-FNS-A score and WTE), a food-level Pearson correlation analysis with familiarity (mean of each protein food) was performed.

No respondents met the exclusion criteria among the 500 follow-up respondents (250 men and 250 women, mean age = 44.81 years, *SD* = 13.96). The test–retest reliability of the J-FNS-A was assessed by calculating the Pearson correlation coefficient of the 8-item J-FNS-A score obtained from the main and follow-up surveys. All statistical analyses were performed using HAD17.202 (70). All tests were two-tailed, and the significance level was set at *p* < 0.05. Effect sizes were reported using the *r* family and interpreted as 0.1 for small, 0.3 for medium, and 0.5 for large, following a previous study (71). In accordance with the sensitivity power analysis, the results are discussed primarily for an effect size of *r* > 0.11.

## 3 Results

### 3.1 Factor structure validity of the J-FNS-A

**Figure 1:**
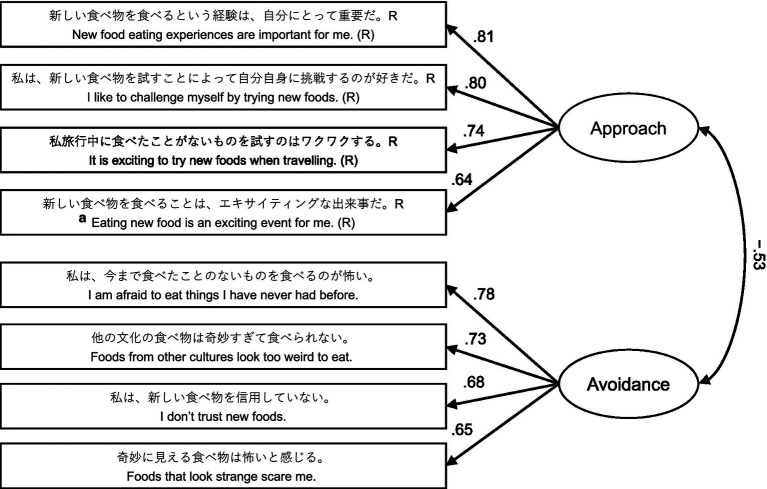
Two-factor model of Japanese version of the alternative food neophobia scale based on confirmatory factor analysis. Rectangles, statements of items (observed variables); ellipses, factors (latent variables). The factor loadings for each item and correlation coefficients between factors are presented. The upper 4 items (statements) in the approach factor reflect food neophilic attitudes, whereas the lower 4 items in the avoidance factor reflect food neophobic attitudes. Item “a” was modified from item 4 (“New food means an adventure for me”) in the original alternative food neophobia scale. All items and two directed question scale items were presented to the participants in random order.

No floor/ceiling effects were observed for any of the 8 items. Correlation coefficients between items within each factor were below *r* = 0.7 (*r*s_approach_ = 0.49–0.68, *r*s_avoidance_ = 0.40–0.55). The validity of the two-factor structural model was tested using CFA, and a CFA-based path diagram is shown in Figure 1. The variance explained by the two factors was 65.3%, and the estimated goodness of fit of the model was satisfactory (GFI = 0.96, CFI = 0.95, RMSEA = 0.087, SRMR = 0.047). The association between two factors (approach and avoidance) was strong (*r* = −0.53). Internal consistencies were all satisfactory (α_approach_ = 0.80, α_avoidance_ = 0.83, α_8-item_ = 0.83).

**Figure 2:**
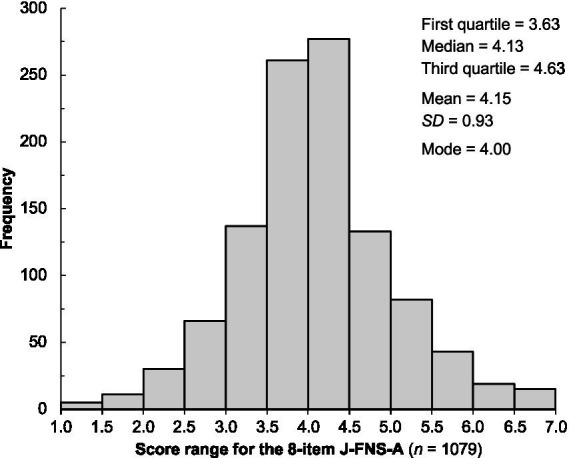
Histogram of Japanese version of the alternative food neophobia scale (J-FNS-A) scores obtained from the main survey (*n* = 1,079).

A histogram of the 8-item J-FNS-A scores is shown in Figure 2. The associations between 8-item J-FNS-A scores and age was not significant (*r* = 0.007, *p* = 0.83). In addition, there was no significant difference between men (mean = 4.13, *SD* = 0.94) and women (mean = 4.17, *SD* = 0.91) in the 8-item J-FNS-A score (*t*[1051.49] = 0.58, *p* = 0.56, *r* = 0.02).

### 3.2 Familiarity with and WTE hamburgers containing various types of protein foods

**Figure 3:**
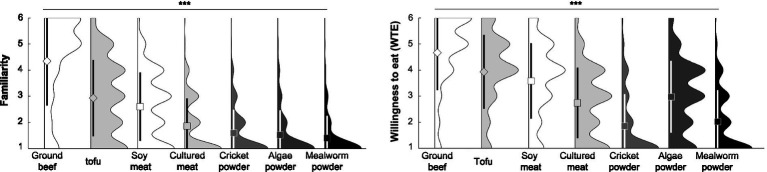
Familiarity and willingness to eat (WTE) score for each protein food. Diamond (◇) or square (□) plot, mean; black or white vertical bold line (error bar), standard deviation (*SD*). The main effect of protein food type and *post-hoc* tests were significant. Detailed results are available in Supplementary Table 2.

The results of familiarity with and WTE hamburgers containing different types of protein foods are shown in Figure 3. More detailed results (measures and statistic value) are shown in Supplementary Table 2. The repeated measures ANOVA for familiarity revealed a significant main effect of protein food type. Post-hoc tests for familiarity were all significant. Familiarity was highest for hamburgers containing ground beef and lowest for hamburgers containing mealworm powder. In addition, there was a significant main effect of protein food type on WTE. Post-hoc tests for WTE were all significant. Although there was small difference in familiarity between cricket and algae powders (*r* = 0.04), WTE algae powder was more than moderately higher than cricket powder (*r* = 0.40). Moreover, correlation analysis of WTE revealed significant associations within protein food types (see Supplementary Table 3). Associations were particularly strong (*r*s > 0.8) between tofu and soy meat, cricket powder, and mealworm powder. In addition, food-level analysis revealed a significant association between familiarity and WTE (*r* = 0.92, *p* < 0.001), suggesting that WTE becomes higher as food familiarity increases.

Further correlation analysis revealed a significant association between WTE and age only for cultured meat, indicating a decrease in WTE with older age (see Supplementary Table 4). In addition, several significant differences between men and women were found for WTE (see Supplementary Table 5). WTE tofu and soy meat was higher among women, whereas WTE cultured meat, cricket powder, and mealworm powder were higher among men.

### 3.3 Predictive validity of the J-FNS-A for WTE alternative protein foods

Correlation analysis was performed to assess the association between J-FNS-A scores and WTE hamburgers with each protein food type (Table 1). Significant associations were found between the J-FNS-A scores (approach, avoidance, and 8-item) and WTE. The J-FNS-A approach score was strongly associated with ground beef and tofu, which have a relatively high familiarity for Japanese people. In contrast, the 8-item J-FNS-A scores were strongly associated with less familiar alternative protein foods (soy meat, cultured meat, cricket powder, algae powder, and mealworm powder).

**Table 1:** Correlation between the 8-item J-FNS-A score and willingness to eat hamburger.

	Patty-protein type	Ground beef	Tofu	Soy meat	Cultured meat	Cricket powder	Algae powder	Mealworm powder
J-FNS-A	Approach	0.27***	0.32***	0.32***	0.31***	0.30***	0.34***	0.32***
	Avoidance	−0.17***	−0.20***	−0.23***	−0.29***	−0.41***	−0.31***	−0.37***
	8-item	−0.26***	−0.31***	−0.33***	−0.35***	−0.42***	−0.38***	−0.41***

Correlation coefficients for the polyserial correlation analysis are shown for each calculation method of J-FNS-A scores. The largest coefficients within the various score calculations are in boldface. Statistical significance is indicated by the *p*-value (***p < 0.001). J-FNS-A, Japanese version of the alternative food neophobia scale.

**Figure 4:**
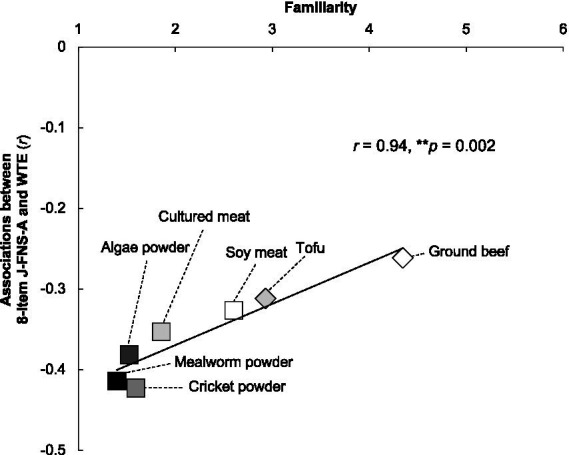
The relation between average familiarity score for each protein food and the correlation coefficients (8-item Japanese version of the alternative food neophobia scale score and willingness to eat) are shown. Statistical significance is indicated by *p*-value (**p < 0.01).

In addition, as a food-level analysis, a correlation analysis was conducted on the associations between (J-FNS-A score, WTE) and familiarity (Figure 4). The results showed that the strength of these associations weakened with increasing familiarity (*r* = 0.94, *p* = 0.002).

### 3.4 Test–retest reliability of the J-FNS-A

The internal consistency of the 8 items was sufficiently high (Cronbach’s α = 0.87). The mean (*SD*) 8-item J-FNS-A scores for the 500 follow-up respondents were 4.17 (0.93) for the main survey and 4.27 (0.99) for the follow-up survey. There was a significant correlation between the 8-item J-FNS-A scores in the main and follow-up surveys (*r* = 0.79, *p* < 0.001).

## 4 Discussion

This study examined the validity of the newly developed J-FNS-A by conducting four web-based surveys among Japanese participants. Factor structure validity, internal consistency, test–retest reliability, and predictive validity were satisfactory for the J-FNS-A. The Japanese version of the FNS-A has demonstrated its potential to quantify FN tendencies in Japanese individuals.

### 4.1 Validities of the J-FNS-A

In preliminary survey 1, 8 items from the original FNS-A were translated into Japanese and tested among Japanese individuals, but 1 item expected to contribute to the approach factor (“New foods mean an adventure for me”) was weakly related to the other 3 items (*r* = 0.13) and required minor wording modifications. This may be explained by the fact that the word “adventure” (“冒険” in Japanese) is not necessarily associated with positive feelings for Japanese respondents. This kind of change in meaning with translation is considered an unavoidable issue when conducting surveys with different national (cultural) groups (53). This may also be related to the fact that the FNS-A was developed primarily using data from relatively young and well-educated South African students (54). Nevertheless, the 8 items of the J-FNS-A in this study were developed using data from a broad age range (20–69 years, 46.8% men and 53.2% women) and a wide range of geographic areas, except Nagano (47), and are sufficiently acceptable for most of the Japanese population.

Furthermore, EFA and CFA are considered important in assessing the validity of a measurement when modifying an existing psychological scale (32). Therefore, CFA was performed on the J-FNS-A, which showed sufficient model fit indices with a two-factor structural model similar to the FNS-A. Contrary to the original FNS (31), which shows a one-factor structural model, numerous FN studies have confirmed a two-factor structure because of differences in the translation and/or cultural backgrounds of respondents (34, 58, 72–75). However, instead of following the CFA results, a previous study (60) noted that the original FNS (31) has proven useful in a massive number of research studies as a single continuous scale and recommend inverting the approach factor score and converting it to an eight-item mean or 10 times the mean (which allows comparison with the original FNS). Indeed, our correlation between the two approach and avoidance factors was large (*r* = 0.53), as in the FNS-A (60), and for internal consistency, the eight items were large enough (α = 0.83). This coincides with the average Cronbach’s alpha coefficient for the four surveys reported previously (60). Furthermore, the internal consistency (Cronbach’s alpha coefficient) of the three surveys (preliminary 2: α = 0.83, main: α = 0.83, follow-up: α = 0.87) was generally higher than the previously reported Japanese translation of Pliner and Hobden’s FNS (45, 47–49), despite including a smaller number of items. The correlations between the 8 items of the J-FNS-A were not very strong (*r*s = 0.09–0.68) considering a single continuous scale, suggesting that each of the 8 items successfully captures various characteristics of FN in Japanese. Accordingly, the main analysis in this study was conducted using the mean of the 8 items (8-item J-FNS-A score). The 1-month test–retest validity of the J-FNS-A score (*r* = 0.79) was almost identical to the 2-week test–retest reliability of the FNS-A (*r* = 0.82) (60).

The mean (*SD*) J-FNS-A score of the 1,079 Japanese participants was 4.15 (0.93). This is significantly higher than the mean scores (ranging from 2.6–2.9) of the three surveys conducted among university students using FNS-A in South African countries (60). In addition, according to De Kock et al. 2022 (60), FNS-A scores can be converted to Pliner and Hobden’s original FN score. The J-FNS-A score (mean = 41.51), multiplied by 10, was notably higher than previous FN scores worldwide (32). Furthermore, this score is higher than the score [*n* = 2,935, mean = 29.99; calculated weighted average by the authors from five studies (33, 34, 44, 76, 77)] obtained by recent studies in East Asian countries (China and Korea). This indicates that only 8.53% of Japanese (92 of 1,079 respondents) are below the East Asian (Chinese and Korean) average. Our result seems to reflect the conservative attitude of Japanese people toward eating novel foods and shows that the FN tendency prevails more in the Japanese population than in other East Asians. However, this tendency may merely reflect the characteristics of the Japanese response style toward the magnitude scale (scale anchors) (78–80) and will not be discussed further in this study. Instead, this study confirmed the association of the J-FNS-A with external factors such as age, gender, and WTE novel foods. According to the results, the 8-item J-FNS-A scores were not related to age (*r* = 0.007) or gender (*r* = 0.02). A consistent relationship between the FN and age has not been reported in previous studies (32). The FN model of defense against unfamiliar/novel foods containing allergens and pathogens suggests that as one ages and acquires knowledge and experience, FN decreases. In fact, it has been reported FN is maximized during childhood and slowly declines during adolescence (30, 72, 81). However, it has been reported that FN tends to increase or remain constant with age (73, 82, 83). This is due to the fact that new, unfamiliar foods are observed daily as a result of globalization in recent years (84), but no specific trend was observed in Japanese participants in the present study. The relationship between gender and FN in Japanese individuals was similar to that previously reported for age. Some reports suggest that women have a weaker FN tendency because they are more involved in purchasing and preparing food (85, 86); however, in general, no gender effects have been observed (32, 87, 88). Given the wide age range and large sample size, our results showed that the FN tendency had almost no association with age and gender in Japanese individuals.

The strength of the association between J-FNS-A scores and WTE hamburgers containing alternative protein foods was above moderate (*r* = −0.42 to −0.33) in this study. This replicates the findings that hamburgers with plant-based patties were tested as unfamiliar foods (60). It also replicates studies using the original FNS (73, 89–91). The strength of the association between the J-FNS-A score and the WTE hamburgers made with alternative protein foods reflects the magnitude of the negative effect of FN on food acceptance. Therefore, by conducting a food-level analysis, the results robustly showed that the effect of FN increases from ground beef hamburgers, with relatively high familiarity, to mealworm hamburgers, whose ingredients are not well known (Figure 4). This finding reflects the concept of FN (28, 31) and clearly explains how exposure to food, rather than its sensory characteristics, is essential for its acceptability (92–94). Overall, we demonstrated that the J-FNS-A predicts individual- and food-level WTE unfamiliar/novel foods, which is consistent with previous studies. In addition, the predictive ability of the J-FNS-A was improved by combining the approach and avoidance factors into a continuous FN tendency rather than separating them (Table 1). This supports further use of the 8-item J-FNS-A calculation method. This study focused on alternative proteins as novel foods for the predictive validation of the J-FNS-A. It has been shown that the strength of the FN tendency is related to the WTE functional and healthy foods (95–97) and the amount of fruits and vegetables consumed (98–102), suggesting that picky eating is associated with FN. The J-FNS-A shows potential not only for future food development but also for helping people make healthier dietary decisions.

This study will make many contributions to future Japanese FN studies, but it also has limitations. The key limitation is that this study did not directly compare the Japanese translation of Pliner and Hobden’s FNS with the J-FNS-A. De Kock et al. proposed the strength of the FNS-A by directly comparing the FNS-A and the modified FNS (almost same as the FNS) with the same respondents to determine the difference in internal consistency and the conversion calculation method for FNS scores. However, this study could not compare the Japanese translation of the FNS with the J-FNS-A because no Japanese translation of the FNS has been reported in previous studies. The J-FNS-A itself has been validated in numerous tests through four surveys and is a sufficiently useful psychological scale. However, for a detailed comparison with the results of previous FN studies conducted worldwide, a direct comparison with the FNS score should be made in the future. This would allow for a comparison of model fit based on CFA, which is more than just an indirect comparison of internal consistency. It is expected that this point will be addressed in future studies.

### 4.2 WTE alternative protein foods

In the process of developing the J-FNS-A, Japanese attitudes toward a variety of alternative protein foods were collected. In this section, we focus on the effects of protein food types on WTE rather than on psychological factors (i.e., FN). Recently, comprehensive research on various alternative protein foods was conducted among 5,000 Japanese individuals (25). Although similar in content, Takeda et al. (25) used alternative protein foods themselves as an example, whereas the difference in this study (*n* = 1,079) was the use of hamburger patties as the main ingredient. Hamburgers are a food item often used in sensory evaluation or alternative protein food studies (22, 60–64). In other words, alternative protein foods are expected to become commonly eaten in the near future. Hence, we primarily discuss the similarities and differences with the study of Takeda et al. (25).

The comparison results among alternative protein foods showed that the WTE hamburger was highest for soy meat, algae powder, cultured meat, and insect powder (crickets and mealworms), in that order, which is consistent with the results of Takeda et al. (25) and those for other countries (13, 21, 23). Our results indicate that the WTE protein substitutes is consistent even when the food recipes are different. However, the WTE hamburger containing soy meat was lower than that for hamburgers containing traditional ground beef or tofu. Tofu is a traditional dietary protein food made from soybeans (103) and is expected to become popular worldwide as an alternative protein food (104). In Japan, it is not traditional to eat tofu as a hamburger patty, but as revealed by food-level analysis, familiarity with the food itself, rather than the food recipes, may have contributed to the greater WTE. In addition, the similarity of food ingredients may also be related to WTE specific alternative protein foods. In this study, strong associations (*r* = 0.82) were found between tofu and soy meat, cricket powder, and mealworm powder for WTE, suggesting that each of these pairs is perceived similarly by Japanese consumers. These similarities among protein foods are conceived to exist since the impressions of alternative proteins are not uniform but are captured as multiple groups (clusters). These similarities are consistent with the study evaluating alternative proteins in terms of the evaluation, potency, and activity dimensions (105), which revealed that the Japanese categorized alternative proteins into plant-based and animal-based sources (106). Moreover, the process of making soybeans into a meat-like food is probably more familiar to the Japanese than to residents of Western countries. There is a type of vegetarian cuisine in Japan called *shojin ryori*, in which soybeans are often used and cooked to resemble foods made with real meat (15). It was originally invented in the 13th century for Japanese Buddhist monks who were forbidden from eating animal-based foods (meat, fish) for religious reasons, and it still draws attention today as a healthy food (107). Furthermore, consistent with Takeda et al. (25), the Japanese appear to have a higher WTE hamburgers containing algae than people from other countries. Specifically, despite a small difference in the familiarity of hamburgers containing cricket powder and algae powder (*r* = 0.04), the WTE hamburger containing algae powder was more than moderately higher than that for the hamburger containing cricket powder (*r* = 0.40). This result deviated from the regression line (*r* = 0.92) between familiarity and WTE in the food-level analysis. According to Takeda et al. (25), this difference in Japanese attitudes toward algae and insects is because respondents associate algae with seaweeds (e.g., nori, wakame, sea lettuce), which they eat in their daily lives as a similar food, even though they are not familiar with the algae themselves. Hence, it is reasonable to assume that even though entomophagy is a traditional practice in certain regions of Japan (47), it is easier for most Japanese individuals today to associate it with strange-looking creatures that exist around them than with food, and thus, the Japanese are not motivated to eat them. The above positive attitudes of the Japanese toward tofu, soy meat, and microalgae were interpreted as evidence that the automatic categorization of foods based on cultural background, such as familiarity with ingredients or making processes (recipes), influences their WTE and/or acceptance.

Furthermore, regarding the gender difference, tofu and soy meat were preferred by women (*r*_tofu_ = 0.15, *r*_soymeat_ = 0.07), whereas men preferred cultured meat (*r* = 0.10) and insect powder (*r*_cricket_ = 0.14, *r*_mealworm_ = 0.08), confirming that plant-based proteins are preferred more by women, similar to the findings of Takeda et al. and other studies conducted in other countries (22, 108). Indeed, from a consumer segmentation perspective, it is important to clarify the relationships between demographic characteristics, such as gender and age, and acceptance (14, 16, 61, 109). However, the difference in gender effects is weaker than that in consumers’ FN effects (*r*s < −0.33). In addition, this study found similar results for WTE for hamburger patty ingredients and ingredients alone. However, this still raises a question about the methodology of this research. This study provided only a brief written description of the alternative protein and hamburger recipe to the participants and did not use any pictures or other information (price, safety, health benefits). This methodological concern implies that this study examined the effect of the quality (impression) of the alternative protein on WTE, rather than the quantity of the alternative protein (percentage of alternative meat replaced by conventional meat). In a recent study examining the consumption of insect-, plant-, and conventional meat-based hamburgers, food information (main composition, eating experience) was found to influence liking, perceived quality, and perceived nutritiousness of the alternative protein food (110). In addition, visual food examples [using food images, serving real food (111)] have the potential to increase the predictive validity of the J-FNS-A by eliciting evaluations (WTE) based on past food experiences. Based on these findings, future studies (surveys and sensory evaluations) investigating the relationship between the J-FNS-A and alternative protein foods will need to carefully consider the methodology of food presentation according to the research question. Given the pre-tasting information (priming) effect, it is expected that there are more ways than food design to motivate consumption of alternative proteins, even among consumers with strong FN.

## 5 Conclusion

In this study, the J-FNS-A was developed and validated through four surveys conducted among Japanese respondents to assess construct validity, internal consistency, test–retest reliability, and predictive validity. The results showed that the J-FNS-A successfully predicted the WTE of unfamiliar foods, using alternative proteins such as insect foods and cultured meat as examples. This study represents the first successful development and validation of an FNS adapted to the Japanese population. The J-FNS-A allows for the quantitative assessment of FN tendencies among Japanese consumers, providing a useful psychological tool for understanding cultural differences in food attitudes and increasing the acceptance of alternative protein foods as well as new foods to be developed soon. Going forward, it is essential to encourage collaboration between researchers developing new foods using novel technology and those exploring eating behavior from a cultural and human sciences perspective. Such collaboration will deepen understanding of consumer food preferences and promote acceptance of novel foods in the Japanese market.

## Data availability statement

The raw data supporting the conclusions of this article will be made available by the authors, without undue reservation.

## Ethics statement

The studies involving humans were approved by the ethics committee of the Food Research Institute, National Agriculture and Food Research Organization, Japan. The studies were conducted in accordance with the local legislation and institutional requirements. The participants provided their written informed consent to participate in this study.

## Author contributions

MK: Data curation, Methodology, Writing – review & editing, Writing – original draft, Investigation, Funding acquisition, Formal analysis, Conceptualization. MN: Writing – review & editing, Software, Formal analysis, Data curation. FH: Writing – review & editing, Validation, Supervision, Investigation. YK: Writing – review & editing, Writing – original draft, Validation, Supervision, Project administration, Conceptualization.
