# Supplementary material for: Development and validation of Japanese version of alternative food neophobia scale (J-FNS-A): association with willingness to eat alternative protein foods
Source: Front Nutr. 2024 May 28;11:1356210. doi: 10.3389/fnut.2024.1356210 (PMC11165137; doi:10.3389/fnut.2024.1356210)
Supplement: Supplementary file 1 [file Table_1.DOCX]

Supplementary Tabl

	Ground beef	Tofu	Soy meat
**Familiarity**	4.35 (1.69)	2.93 (1.45)	2.60 (1.30)
**Willingness to eat (WTE)**	4.66 (1.43)	3.94 (1.41)	3.59 (1.44)

Supplementary Table 2. Repeated measures ANOVA results

	Mean (*SD*)									
	Ground beef	Tofu	Soy meat	Cultured meat	Cricket powder	Algae powder	Mealworm powder	*F*	*p*	η^2
**Familiarity**	4.35 (1.69)	2.93 (1.45)	2.60 (1.30)	1.86 (1.05)	1.59 (0.89)	1.52 (0.94)	1.40 (0.85)	1267	< 0.001	0.54
**Willingness to eat (WTE)**	4.66 (1.43)	3.94 (1.41)	3.59 (1.44)	2.74 (1.35)	1.85 (1.22)	2.97 (1.37)	2.02 (1.21)	1138	< 0.001	0.51

results

t	*p*	*r*
1.09	0.28	0.03
4.81	< 0.001	0.15
2.24	0.025	0.07
3.21	0.001	0.10
4.62	< 0.001	0.14
0.16	0.88	0.005
2.75	0.006	0.08

Supplementary Ta

		Ground beef
**Willingness to eat (WTE)**	Ground beef	1
	Tofu	0.55***
	Soy meat	0.49***
	Cultured meat	0.35***
	Cricket powder	0.13***
	Algae powder	0.35***
	Mealworm powder	0.08*

Supplementary Table 2. Repeated measures A

Mean (*SD*)					
Ground beef	Tofu	Soy meat	Cultured meat	Cricket powder	Alg pow

Supplementary Table 2. Repeated measures ANOVA results

Mean (*SD*)								
Ground beef	Tofu	Soy meat	Cultured meat	Cricket powder	Algae powder	Mealworm powder	*F*	*p*
4.35 (1.69)	2.93 (1.45)	2.60 (1.30)	1.86 (1.05)	1.59 (0.89)	1.52 (0.94)	1.40 (0.85)	1267	< 0.001
4.66 (1.43)	3.94 (1.41)	3.59 (1.44)	2.74 (1.35)	1.85 (1.22)	2.97 (1.37)	2.02 (1.21)	1138	< 0.001

Note: Statistical signifficance was indicated by the p-value (***
